# Segmented golden ratio radial reordering for dynamic cardiac MRI with variable temporal resolution

**DOI:** 10.1186/1532-429X-17-S1-Q124

**Published:** 2015-02-03

**Authors:** Fei Han, Ziwu Zhou, Stanislas Rapacchi, Paul J Finn, Peng Hu

**Affiliations:** 1 Radiological Sciences, David Geffen School of Medicine at UCLA, Los Angeles, CA, USA; 2 Department of Bioengineering, University of California, Los Angeles, Los Angeles, CA, USA; 3 Biomedical Physics Inter-Departmental Graduate Program, University of California, Los Angeles, Los Angeles, CA, USA

## Background

**Figure 1:**
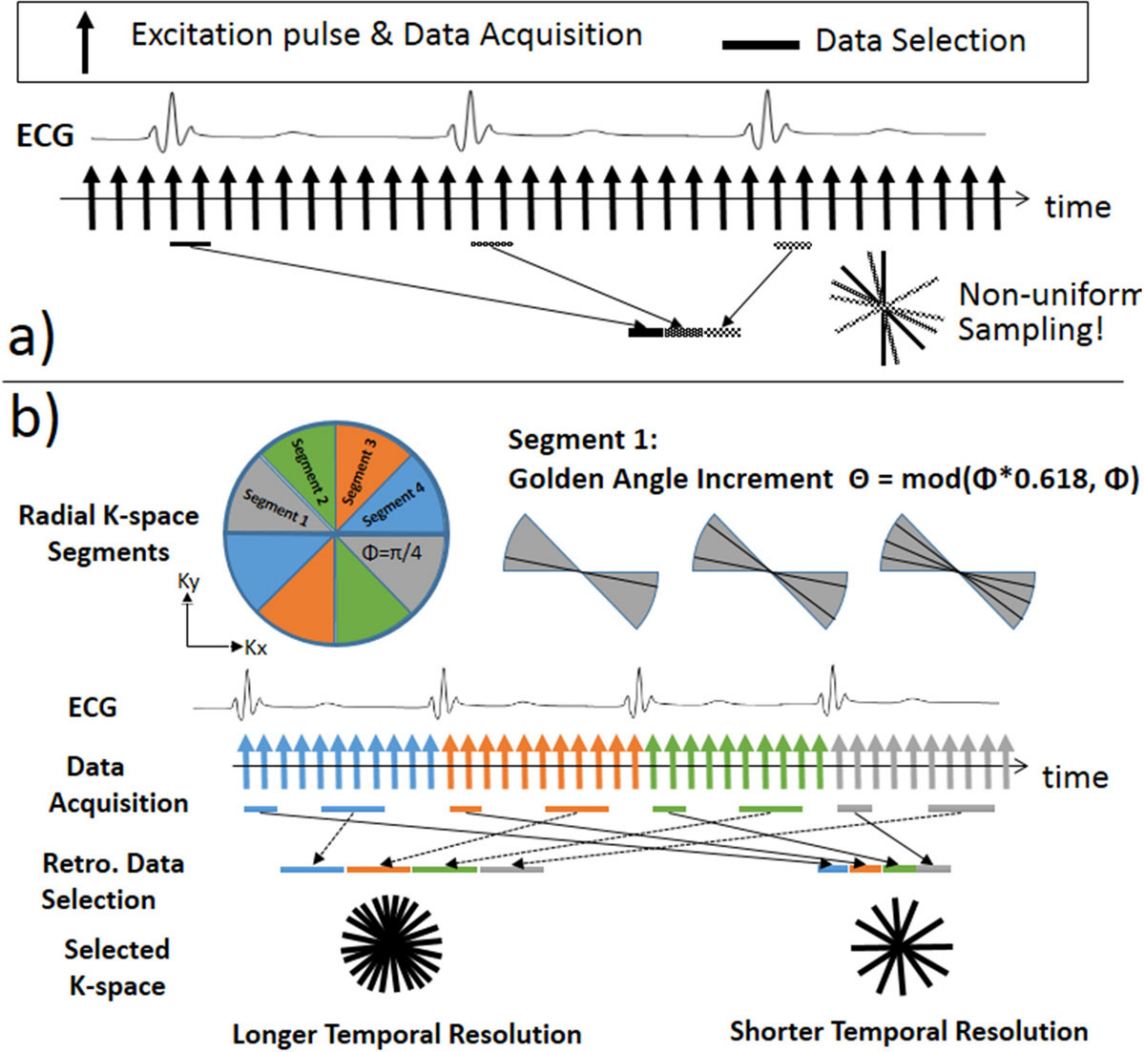
a) Conventional GA method, when applied to ECG-gated cardiac imaging applications, results in non-uniform sampling of k-space; b) The proposed segmented GA method addresses this issue by performing golden-ratio acquisition inside each segment within a R-R interval and thus provides a near-uniform k-space sampling of arbitrary acquisition window length and position within a cardiac cycle.

Golden angle radial reordering (GA) has been applied in many abdominal and neurological imaging applications to allow for retrospective choice of temporal resolution by providing a near-uniform k-space sampling within any image reconstruction time window [1]. However, its application in cardiac imaging is limited because the ECG-gated acquisition, which is required in most cases, breaks a single reconstruction window into several temporally isolated k-space data so that the k-space coverage may not be as uniform as GA without ECG gating (Fig. 1a) [2]. Therefore, we sought to investigate the image artifacts caused by applying GA to ECG-gated cardiac imaging and propose a segmented GA method to address this issue.

## Methods

The proposed segmented GA method calculates the azimuthal angle based on the total number of k-space segments S and the current segment index n (Eq.1). In the example shown in Fig. 1b, there are 4 k-space segments and each one was acquired within one of the 4 ECG-RR intervals. Since each segment is acquired in a golden-ratio manner, any subset of these measurements forms a near-uniform sampling of the current segment and together with other segments, forms a near-uniform sampling of the entire k-space.

a_k+1_=mod[(a_k_+(π/S)*0.618), (π/S)] + nπ/S, a_0_=0; (Eq.1)

Computer simulations based on a Shepp-Logan phantom were performed to compare the k-space sampling pattern of conventional non-ECG-gated GA, ECG-gated GA and the segmented GA (12 simulated heartbeats, 100-120 radial views in each heartbeat, views for reconstruction N=60, 96, 192 and 240). In-vivo 2D radial and 3D "stack-of-stars" breath-hold, ECG-gated cardiac CINE were acquired on 8 healthy volunteers using both ECG-gated GA and segmented GA (1.5T; bSSFP; 1.6 /3.3ms; FA=50; 1.3x1.3x6mm^3^).

## Results

**Figure 2:**
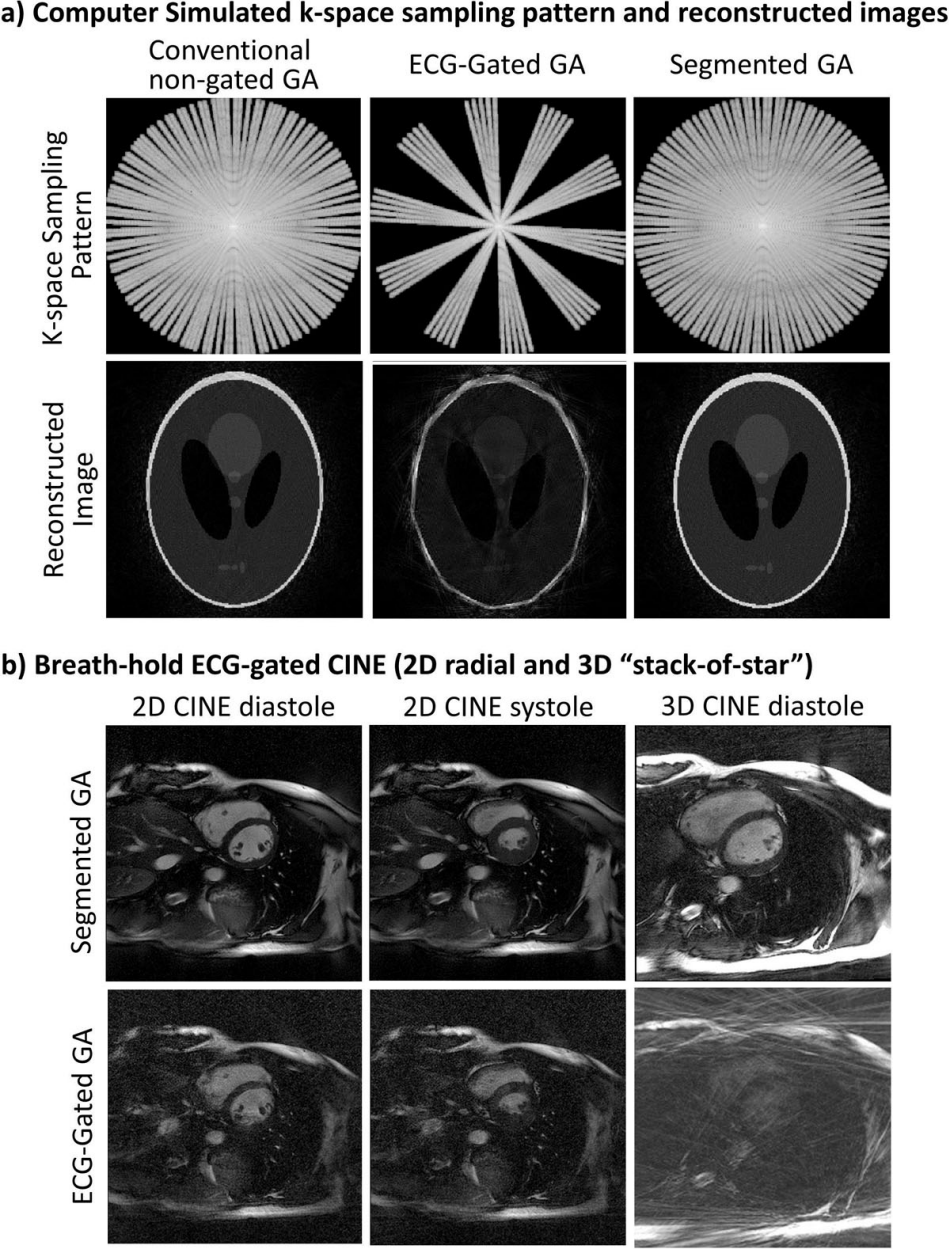
a) Computer simulation results of conventional non-ECG-gated GA, ECG-gated GA and the segmented GA. Both the non-ECG-gated GA and the segmented GA provide near-uniform sampling pattern and reconstructed images with good quality while the reconstructed image in ECG-gated GA case has streaking artifacts due to poor sampling uniformity. b) For both 2D (216 radial views, temporal resolution 48ms) and 3D (144 views, 120ms), the proposed segmented GA approach offers much improved image quality than ECG-gated GA even though same number of radial views were used for reconstruction.

Fig. 2a shows the simulated k-space sampling patterns and reconstructed images (N=60). Both the segmented GA and conventional GA provided a near-uniform k-space sampling pattern and good image quality. However, the ECG-gated GA image had streaking artifacts due to poor sampling uniformity. The in-vivo 2D CINE images shown in Fig. 2b were reconstructed using 216 views with temporal resolution of 48ms. Based on 8 volunteer's data, the segmented GA method provided images with higher SNR and less streaking artifacts compared to ECG-gated GA (blood pool SNR: 6.4±0.3 vs 3.4±0.7 P<0.05). The 3D CINE images using ECG-gated GA (144 views, temporal resolution 120ms) in Fig. 2b was non-diagnostic due to strong streaking artifacts while the one using segmented GA provided much better image quality even though same number of radial views were used.

## Conclusions

The proposed segmented GA method successfully addresses the non-uniform sampling issue when combining conventional GA with ECG gating and can be potentially applied to any ECG-gated cardiac imaging applications, including cine and flow imaging, to allow retrospective selection of temporal resolution.

## Funding

N/A.
